# Evidence that *p53*-Mediated Cell-Cycle-Arrest Inhibits Chemotherapeutic Treatment of Ovarian Carcinomas

**DOI:** 10.1371/journal.pone.0000441

**Published:** 2007-05-16

**Authors:** Carlos S. Moreno, Lilya Matyunina, Erin B. Dickerson, Nina Schubert, Nathan J. Bowen, Sanjay Logani, Benedict B. Benigno, John F. McDonald

**Affiliations:** 1 Department of Pathology and Laboratory Medicine, Emory University School of Medicine, Atlanta, Georgia, United States of America; 2 Winship Cancer Institute, Atlanta, Georgia, United States of America; 3 School of Biology, Georgia Institute of Technology, Atlanta, Georgia, United States of America; 4 Ovarian Cancer Institute, Atlanta, Georgia, United States of America; University of Minnesota, United States of America

## Abstract

Gene expression profiles of malignant tumors surgically removed from ovarian cancer patients pre-treated with chemotherapy (neo-adjuvant) prior to surgery group into two distinct clusters. One group clusters with carcinomas from patients not pre-treated with chemotherapy prior to surgery (C-L), while the other clusters with non-malignant adenomas (A-L). We show here that although the C-L cluster is preferentially associated with *p53* loss-of-function (LOF) mutations, the C-L cluster cancer patients display a more favorable clinical response to chemotherapy as evidenced by enhanced long-term survivorships. Our results support a model whereby *p53* mediated cell-cycle-arrest/DNA repair serves as a barrier to optimal chemotherapeutic treatment of ovarian and perhaps other carcinomas and suggest that inhibition of p53 during chemotherapy may enhance clinical outcome.

## Introduction

Ovarian cancer is the most deadly of gynecologic malignancies and the fourth leading cause of all cancer deaths of women in the United States [1]. Because the disease is essentially asymptomatic early in its progression, approximately 70% of all ovarian cancers are not diagnosed until advanced stages (FIGO stage III or IV) when long-term prognosis is poor (<20% long-term survival) [2]. The current standard treatment for patients with advanced ovarian cancer is cytoreductive surgery followed by platinum/taxane combination therapy [3]. While this treatment can be effective in the short-term, 80% of patients relapse within 5 years. The failure of current therapies to significantly improve the long-term survivorship is believed to be due primarily to the development of chemotherapy resistance, e.g. [4], [5].

In recent years, significant effort has focused on the identification of molecular markers that can predict the likely response of ovarian cancer patients to chemotherapeutic treatments with the ultimate goal of developing optimal treatments for individual patients. An experimental approach successful in predicting the outcome of chemotherapy treated patients is gene expression profiling [e.g. 6]–[8]. Several such profiling experiments have been carried out on ovarian tumor samples removed from patients prior to chemotherapy treatment [e.g. 9], [10]. Collectively, these studies indicate that gene expression profiling of ovarian and other cancers holds significant promise, not only as prognostic indicators of clinical outcome, but as a means of identifying specific molecular abnormalities that may underlie various manifestations of the disease.

Here, we report on a gene expression profile analysis of ovarian carcinoma samples obtained after neo-adjuvant chemotherapy (carboplatin/taxol), samples from primary surgical resections, and non-malignant ovarian adenoma tissues (Table 1). Our results demonstrate that the gene expression profiles of the primary carcinomas and non-malignant adenomas cluster into two distinct groups. The neo-adjuvant treated patient samples cluster with either the primary carcinoma samples or with the non-malignant adenomas. The neo-adjuvant samples that clustered with the primary carcinomas were preferentially associated with LOF mutations in the *p53* gene and displayed an expression profile characteristic of a highly proliferative state. Comparison of our expression profiles with the previously established “Ovarian Cancer Prognostic Profile” (OCPP) [6] demonstrated a significant overlap in profiles, and predicted a more favorable outcome for patients whose samples clustered with the primary carcinomas. Survivorship profiles of patients involved in our study were found to be consistent with this prediction. Our findings indicate that *p53* mediated cell-cycle-arrest/DNA repair serves as a barrier to optimal chemotherapeutic treatment of ovarian and perhaps other carcinomas and suggest that inhibition of *p53* during chemotherapy may enhance the long-term survivorship of ovarian cancer patients.

**Table 1:** Patient samples analyzed in this study.

Cluster	Sample	Malignant Potential	Histological Information	Stage/Grade	Age at surgery
A-L					
	AD64	benign	Serous cystadenofibroma	na	84
	AD77	benign	Serous cystadenofibroma	na	51
	AD97	benign	Serous cystadenofibroma	na	61
	AD125	benign	Serous cystadenoma	na	61
	AD132	benign	Serous cystadenoma	na	47
	AD146	benign	Simple cystadenoma	na	74
	AD159	benign	Simple cystadenoma	na	70
	AD172	benign	Simple cystadenoma	na	61
	AD221	benign	Simple cystadenoma	na	67
	AD300	benign	Serous cystadenofibroma	na	71
	CC9	invasive malignant	Serous papillary adenocarcinoma	IV/2	51
	CC36	invasive malignant	Serous papillary adenocarcinoma	III/3	66
	CC150	invasive malignant	Serous papillary adenocarcinoma	IIIc/3	65
	CC184	invasive malignant	Serous papillary adenocarcinoma	III/3	67
	CC279	invasive malignant	Serous papillary adenocarcinoma	IIIb/3	62
	CC286	invasive malignant	Serous papillary adenocarcinoma	IIIc/2	52
	CC303	invasive malignant	Serous papillary adenocarcinoma	IIIc/3	44
	CC310	invasive malignant	Serous papillary adenocarcinoma	IV/2	41
	CC311	invasive malignant	Serous papillary adenocarcinoma	IIIc/2	51
	CC314	invasive malignant	Serous papillary adenocarcinoma	III/2	79
	CC325	invasive malignant	Serous papillary adenocarcinoma	IIIc/2	75
	CC326	invasive malignant	Serous papillary adenocarcinoma	IV/3	72
	CC338	invasive malignant	Serous papillary adenocarcinoma	IIIc/3	62
C-L					
	CA2	invasive malignant	Serous papillary adenocarcinoma	IIb/3	61
	CA4	invasive malignant	Serous papillary adenocarcinoma	IIIb/3	48
	CA23	invasive malignant	Serous papillary adenocarcinoma	IIIa/3	51
	CA66	invasive malignant	Serous papillary adenocarcinoma	IV/3	74
	CA99	invasive malignant	Serous papillary adenocarcinoma	III/3	75
	CA183	invasive malignant	Serous papillary adenocarcinoma	III/2	66
	CA196	invasive malignant	Endometriod adenocarcinoma	III/2	45
	CA204	invasive malignant	Endometriod adenocarcinoma	Ic/3	47
	CA212	invasive malignant	Serous papillary adenocarcinoma	IIIc/3	59
	CC29	invasive malignant	Serous papillary adenocarcinoma	III/3	66
	CC76	invasive malignant	Serous papillary adenocarcinoma	IIIa/2	49
	CC94	invasive malignant	Serous papillary adenocarcinoma	IIIc/1	55
	CC187	invasive malignant	Serous papillary adenocarcinoma	IIIc/2	53
	CC199	invasive malignant	Serous papillary adenocarcinoma	IIIc/3	69
	CC253	invasive malignant	Endometriod adenocarcinoma	IIIc/3	56
	CC255	invasive malignant	Undifferentiated carcinoma	IIIc/2	57
	CC259	invasive malignant	Serous papillary adenocarcinoma	IIIb/3	58
	CC269	invasive malignant	Serous papillary adenocarcinoma	IIIc/3	68
	CC272	invasive malignant	Serous papillary adenocarcinoma	IIIb/2	83
	CC312	invasive malignant	Serous papillary adenocarcinoma	IIIc/2	64

## Results

### Gene expression profiles of ovarian carcinomas from neo-adjuvant patients cluster into two groups

**Figure 1:**
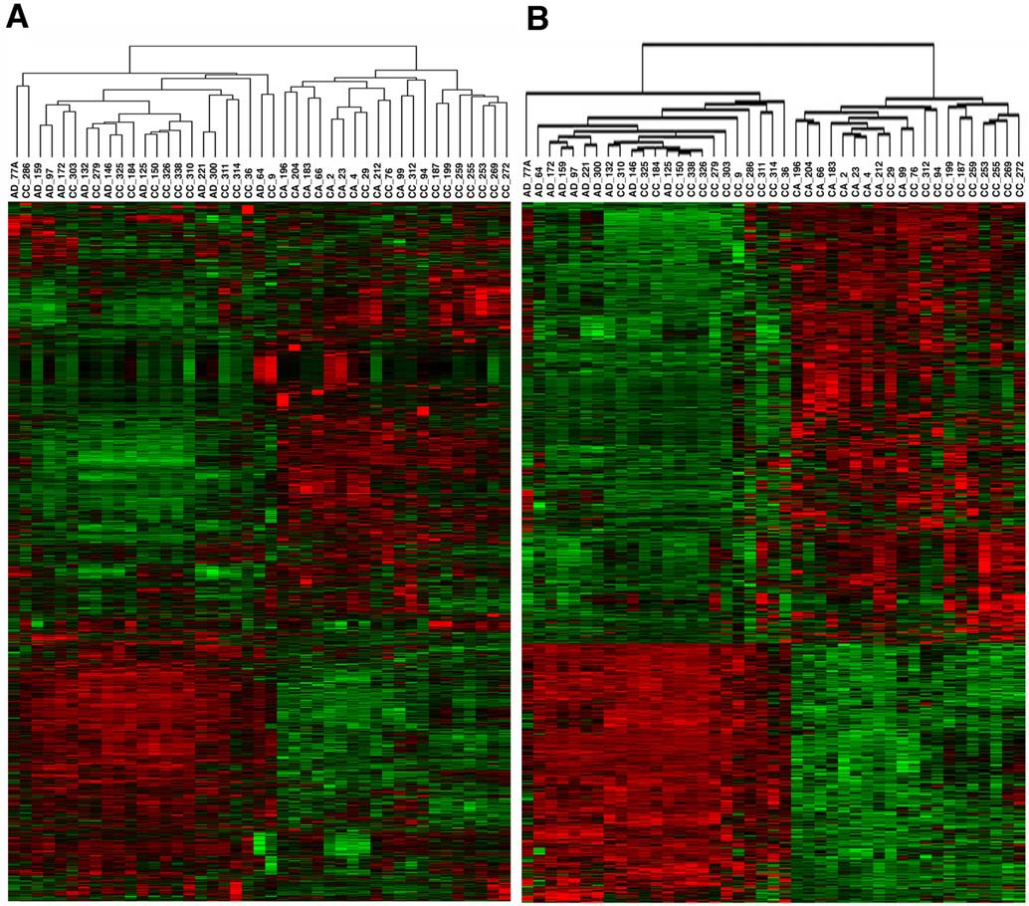
(A) Unsupervised hierarchical clustering of the gene expression pattern of all 9,106 expressed probe sets detected on the HG-U95Av2 GeneChip in 43 ovarian tumor samples. Samples beginning with AD are adenomas, with CA are carcinomas, and CC are patients pre-treated with chemotherapy. Samples divided into two major clusters termed adenoma-like and carcinoma-like. (B) Hierarchical clustering of the 1,527 probe sets that were significantly altered (>2-fold, FDR<1%) between the carcinoma-like and adenoma-like samples.

Unsupervised clustering of the expression profiles of all of the genes detected on the Affymetrix HG_U95Av2 GeneChip microarrays was performed on ovarian adenomas (AD) and carcinomas from neo-adjuvant (CC) and untreated (CA) patients. This initial unsupervised analysis was performed using average linkage of Euclidian distance on all 9,106 probe sets in which the signal from at least one sample exceeded an arbitrary threshold (signal≥32). The resulting clustering pattern (Figure 1A) divided the samples into two main groups. The carcinomas (CA) and adenomas (AD) from patients not receiving chemotherapy formed two distinct and separate clusters, while the neo-adjuvant treatment carcinomas (CC) were divided equally between these two groups. These two groups were designated “carcinoma-like” (C-L) and “adenoma-like” (A-L) respectively. These two main clusters were very robust and were not changed if the samples were re-ordered, or if the samples were clustered using alternative metrics such as Manhattan city-block, uncentered correlation, or Spearman Rank correlation.

To identify a signature of genes that were significantly different between the A-L and C-L group, we performed supervised analysis using the Significance Analysis of Microarrays (SAM) software [11]. Using thresholds of 2-fold change and<1% False Discovery Rate (FDR), 1,527 probe sets corresponding to 1,334 unique genes were found to be significantly (p<0.05) different between the C-L and A-L sample groups (Figure 1B). A heat map giving the names of each of the 1,527 probe sets is available in the supplementary material (Figure S1).

**Figure 2:**
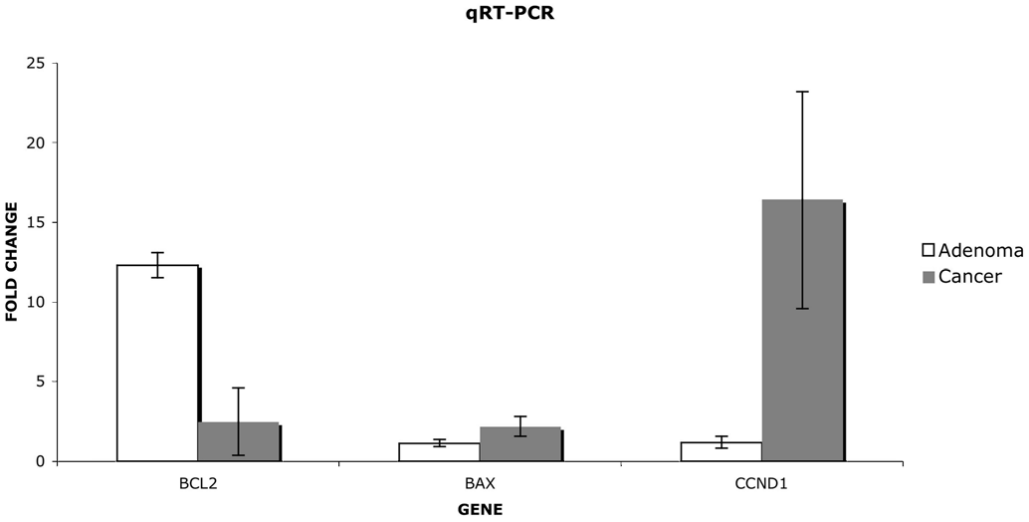
qRT-PCR validation of microarray results. The expression patterns of BCL2, BAX and CCND1 in 4 tissue samples were determined using quantitative RT-PCR. In all cases, the qRT-PCR results of the analyses confirmed the differences detected in the microarray studies.

To independently test the validity of the differential expression patterns determined by microarray, we measured the expression patterns of 3 representative genes in 4 tissue samples using quantitative real-time polymerase chain reaction (qRT-PCR). In all cases, the qRT-PCR results of the analyses confirmed the differences detected in the microarray studies (Figure 2).

### Gene Ontology (GO) analysis identifies functional differences between the C-L and A-L groups

To determine if the 1,334 genes significantly altered between C-L and A-L groups were enriched for specific biological functional categories, we analyzed this gene set for enrichment in GO functional annotations using GOstat [12]. Several functional categories were significantly (p<0.05) over-represented in this gene set relative to the total GO categories available on the U95Av2 GeneChip (Table 2). Most notable were biological functions involved in the cell cycle, cellular proliferation, cell cycle checkpoints, stress response, DNA damage, and apoptosis. In general, the C-L group demonstrated increased expression of genes involved in nucleosome assembly, including histone genes, suggestive of a higher proportion of cells in S phase.

**Table 2:** GO annotations significantly enriched in genes altered between A-L and C-L groups.

*GO*	*Significant Genes*	*Total Genes*	*Pvalue*	*GO as name*
GO:0006955	141	470	2.02E-18	immune response
GO:0000786	16	18	2.89E-09	nucleosome assembly
GO:0007155	103	381	2.89E-09	cell adhesion
GO:0006950	130	621	0.000834	response to stress
GO:0007051	10	16	0.00192	spindle organization and biogenesis
GO:0000074;				
GO:0007049	77	320	0.00192	regulation of cell cycle
GO:0000785	25	77	0.00241	chromatin
GO:0000278	39	143	0.00355	mitotic cell cycle
GO:0008632	13	29	0.00872	apoptotic program

The p-value for GO significance was computed with GOstat (12) using the Benjamini FDR correction for multiple testing and the HG_U95Av2 gene collection for comparison. Significant genes indicates the number of genes significantly altered between A-L and C-L tumors with the given GO annotation. Total genes indicates the total number of genes with that GO annotation on the HG_U95Av2 GeneChip.

### Predictive models accurately distinguish between the C-L and A-L sample groups

**Figure 3:**
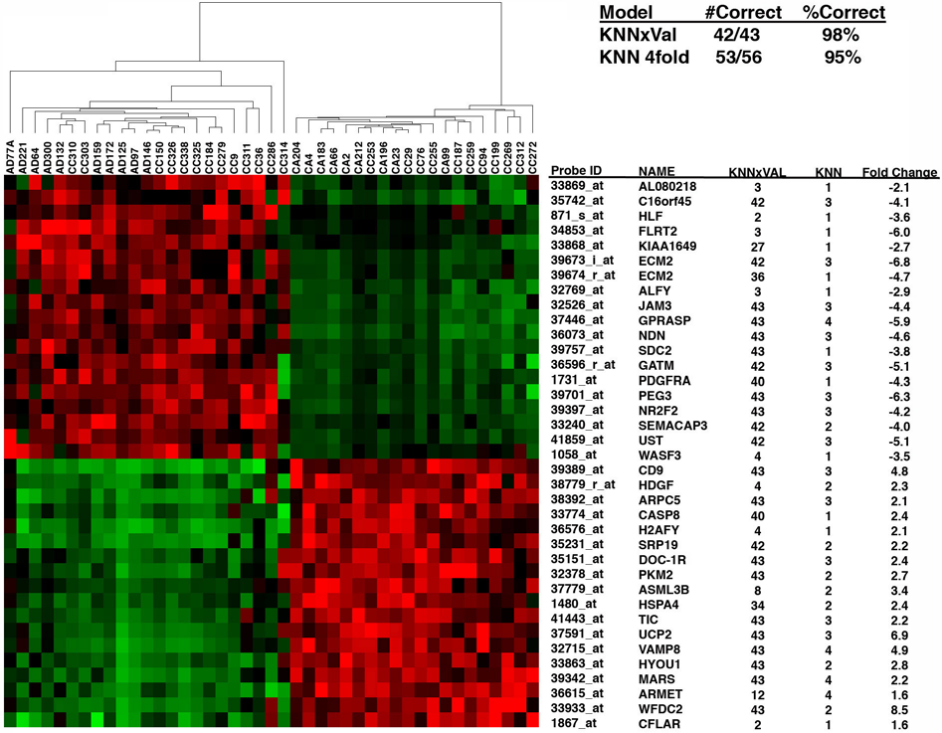
Two-dimensional hierarchical clustering of 43 samples is shown using a set of 36 probe sets that were employed in at least one KNN predictive model and two or more KNNxVal models. To the right of the clustering pattern are given the probe set ID, HUGO Gene Symbol, number of models that each probe set appeared, and the fold change for each probe set for carcinoma-like vs. adenoma-like samples. Also shown are the number and percent correct predictions made by the 43 KNNxVal models and the four KNN models (upper right panel).

To determine if we could use the expression profiles from these samples to identify sets of genes that would be predictive of the C-L and A-L sample clusters, we used both K- nearest neighbor leave-one-out cross validation (KNNxVal) and KNN randomized four-fold cross validation methods. For both methods, we built models using sets of the 30 genes to predict whether “unknown” samples partitioned to one group or the other. Using the KNNxVal approach, we built 43 models and correctly predicted the sample class in 42/43 (99%) of the cases. For the KNN approach, the samples were randomly divided into training sets of 29 cases, and test sets of 14 cases. Models were built using the 29 cases in the training sets and subsequently used to predict the grouping (class) of the 14 test cases. For these models, the class of the predicted samples was correctly determined in 95% of the cases. Because many predictor models were generated (43 KNNxVal and 4 KNN models), we identified the 37 most predictive genes as those that were used in at least two of the KNNxVal models and were used in at least one of the KNN models. The expression profiles, identities, fold changes, and the number of models in which these probe sets were used, are presented in Figure 3.

Prediction of CC samples into C-L and A-L clusters was also achieved with 100% accuracy by support vector machine (SVM) analysis using GenePattern software.

### Altered biological pathways characterize the C-L and A-L groups

**Figure 4:**
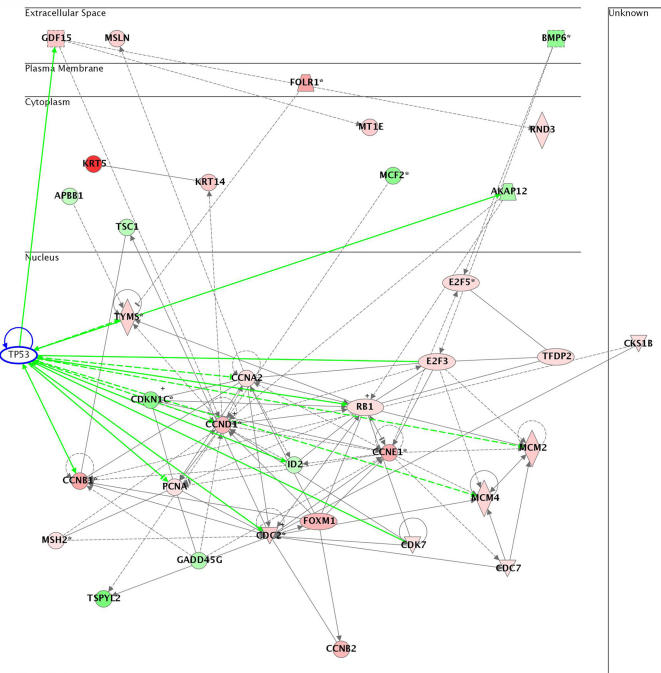
Gene interaction network of altered genes associated with cell cycle regulation and cancer as determined by Ingenuity Pathways Analysis. Genes colored red showed increased expression in carcinomas relative to adenomas and in green had decreased expression in carcinomas relative to adenomas. Increased levels of cyclin D1 (CCND1), cyclin E1 (CCNE1), cyclin B1 (CCNB1), cyclin B2 (CCNB2), cyclin A2 (CCNA2), E2F3, E2F5, cyclin-dependent kinases (CDC2 and CDK7) as well as decreased levels of p57 cyclin-dependent kinase inhibitor (CDKN1C) and growth arrest and DNA-damage-inducible, gamma (GADD45G) are all consistent with carcinomas having higher proliferation rates than adenomas. Interactions of these genes with p53 are highlighted as green lines.

**Figure 5:**
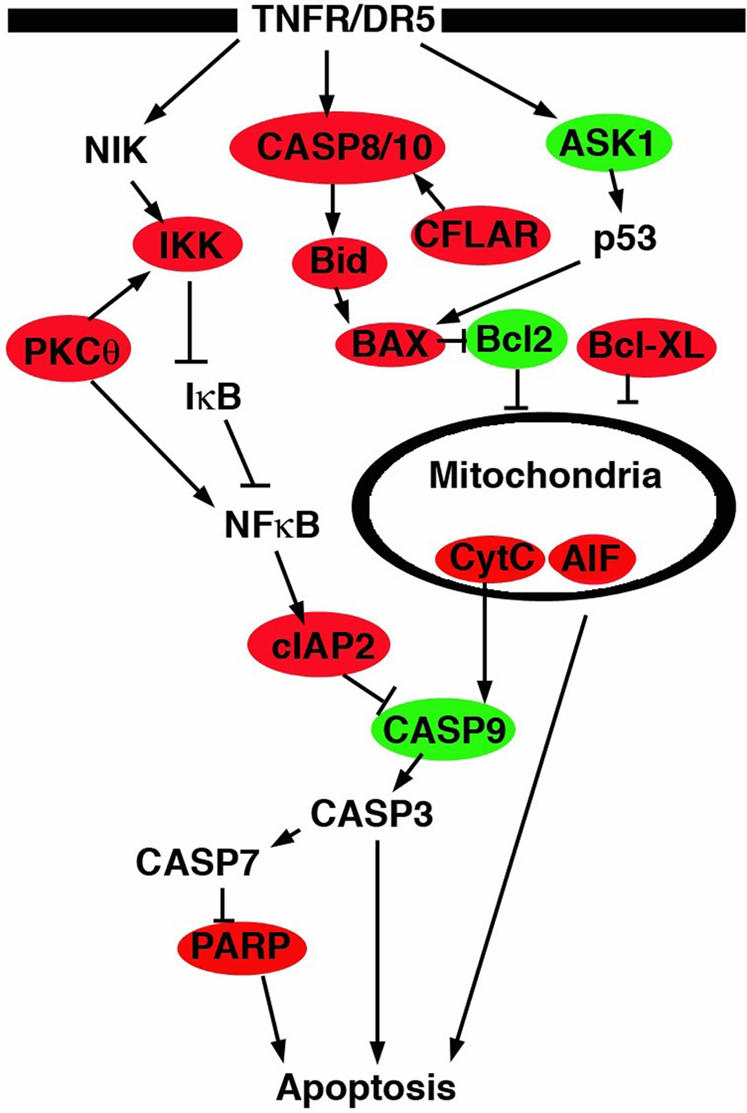
Apoptosis pathway with genes displaying increased expression (colored red) and decreased expression in carcinoma-like samples (colored green). Carcinoma-like samples had higher expression of pro-apoptotic genes such as BAX, BID, CASP8, and AIF. These samples also expressed higher levels of anti-apoptotic factors such as IKK, Bcl-xl, and c-IAP2. Adenoma-like samples had higher Bcl-2 expression than carcinoma-like samples.

To investigate biological pathways possibly affected by the highly predictive genes, we performed Ingenuity Pathway Analysis (IPA) (http://www.ingenuity.com/) on the set of the 37 most predictive genes used in our predictive models (see above). A number of pathways were found to be affected by the genes that were predictive between the C-L and A-L groups (Figure 4). Among the most significant pathways was the death receptor apoptosis pathway (p<0.01). Figure 5 shows a simplified canonical Death Receptor Pathway, with genes increased in the C-L group colored in red, and genes decreased in C-L group colored in green.

### C-L group samples are associated with *p53* LOF mutations

**Figure 6:**
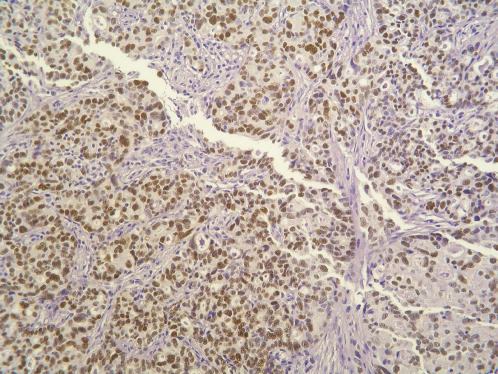
p53 immunohistochemical staining of a serous papillary adenocarcinoma (sample CA99) displaying strong diffuse p53 immunoreactivity. Intense staining of tumor cells is typically associated with the presence of point mutational damage in *p53* exons 5–8 (magnification X 400).

Cisplatin is a DNA damaging agent that is expected to trigger *p53*-dependent cell cycle arrest and apoptosis in tumors with wild-type *p53* tumor suppressor function. Several of the GO categories enriched for genes that differed between the C-L and A-L expression profile groups relate directly to *p53* function. For example, cell cycle regulation, stress response, DNA repair, and apoptosis are all profoundly influenced by the status of *p53* [13], [14]. Downstream targets of *p53* that were altered between these groups were identified using Ingenuity Pathway Analysis software. A summary of the observed mRNA fold change between the C-L and A-L samples of genes recognized as downstream transcriptional targets of *p53* is given in Table 3. Of the downstream targets altered between the C-L and A-L groups, 16/23 (70%) displayed changes in gene expression consistent with a loss of *p53* function in the C-L group. Since we detected no significant difference in *p53* expression among the samples examined in this study, we explored the possibility that *p53* LOF (loss-of-function) mutations were preferentially associated with the C-L group. Tissue samples from both the A-L and C-L cluster groups were assayed for immunohistochemical staining using a *p53* antibody (Figure 6). Positive staining has been previously employed as an indicator of *p53* mutations [26]. In our study, 4 out of the 6 positively staining samples were correlated with *p53* LOF mutations (see below) while 11 out of the 16 negatively staining samples were correlated with no or functionally equivalent mutations (Table 4). Direct testing for *p53* LOF mutations was carried out by DNA sequencing. cDNA was synthesized from mRNA isolated from 14 CC, 5 CA and 6 AD samples. Regions of the *p53* gene (exons 5-10) previously associated with LOF mutations [27], [28] were amplified using PCR and sequenced (Table 4). Consistent with previously published results [29], we found that about half of ovarian cancer samples from patients not pre-treated with chemotherapy prior to surgery are associated with *p53* LOF mutations. In contrast, only 1 of the 6 AD samples we examined was associated with known *p53* LOF mutations. The 7 CC samples clustering within the A-L group had either no mutations or were associated with functionally silent mutations in *p53* gene. Remarkably, 6 of the 7 tested CC samples whose expression profiles clustered within the C-L group were associated with missense or non-sense mutations and 5 of these have previously been associated with loss of *p53* function. Analysis of the correspondence of *p53* LOF mutations with the C-L and A-L clusters was significant (p = 0.006 by Fisher's Exact Test). These results support the hypothesis that the functional status of *p53* is a major distinguishing characteristic between the neo-adjuvant (CC) samples clustering in the C-L and A-L groups.

**Table 3:** Changes in expression of known *p53* target genes between A-L and C-L group tumor samples.

*Target Gene*	p53 *effect*	*Fold change* *Including CC*	*Fold change CA/AD only*	*Consistent with p53 mutation*	*Reference*
MMP9	Repressed	11.3	27.7	Y	[13]
STK6	Repressed	5.1	8.4	Y	[14]
ARPC1B	Repressed	4.1	5.5	Y	[14]
CDC2	Repressed	4.0	6.5	Y	[15]
PDE4B	Repressed	3.0	2.9	Y	[14]
HMMR	Repressed	2.6	3.8	Y	[14]
RB1	Repressed	2.5	3.7	Y	[16]
PCNA	Repressed	2.1	3.0	Y	[17]
ID2	Induced	−2.1	−3.0	Y	[14]
PPM1D	Induced	−2.2	−2.0	Y	[18]
CAV1	Induced	−2.6	−3.5	Y	[19]
GSTM1	Induced	−3.1	−5.1	Y	[20]
FDXR	Induced	−3.1	−6.8	Y	[21]
COL14A1	Induced	−3.9	−15.6	Y	[20]
FHL2	Induced	−4.3	−6.0	Y	[22]
PEG3	Induced	−6.3	−39.3	Y	[23]
SLPI	Induced	6.7	9.0	N	[20]
S100A2	Induced	5.1	5.2	N	[24]
IRF5	Induced	2.8	2.5	N	[25]
AKAP12	Repressed	−2.9	−3.2	N	[14]
ENG	Repressed	−3.7	−7.4	N	[14]
HS3ST1	Repressed	−6.5	−9.1	N	[14]

Sixteen of 23 *p53* target genes (69.6%) were altered in a manner consistent with a loss of p53 function in ovarian carcinomas.

**Table 4:** Summary of immunohistochemistry staining (IMMUNO HIS) and mutations found in the *p53* gene in tumor samples from patients not pre-treated with chemotherapy prior to surgery (A) and neo-adjuvant treated patients (B).

A.				
CLUSTER	PATIENT	IMMUNO HIS	MUTATION	ACTIVITY
C-L	CA99	+	C176F	LOF
"	CA183	nd	D259D	Silent
"	CA196	+	G389G	Silent
"	CA204	+	C274Y	LOF
"	CA212	-	none	Functional
A-L	AD125	-	none	Functional
"	AD132	nd	K139E	Functional
"	"		E221V	Functional
"	"		P300P	Silent
"	"		A364A	Silent
"	AD146	-	none	Functional
"	AD159	-	none	Functional
"	AD172	-	none	Functional
"	AD300	-	none	Functional

B.				
CLUSTER	PATIENT	IMMUNO HIS	MUTATION	ACTIVITY
C-L	CC187	+	P151R	LOF
“	“		R156C	LOF
“	CC199	+	I162I	Silent
“	“		N236D	LOF
“	CC255	+	N131N	Silent
“	“		C135R	LOF
"	CC259	-	A159A	Silent
“	“		N200S	Functional
“	“		R333R	Silent
“	“		Q336V	Functional
“	CC269	-	N131N	Silent
“	“		K139Q	LOF
“	“		L252P	LOF
“	“		S313G	Functional
“	CC272	+	I195I	Silent
“	“		R282W	LOF
“	CC312	-	T150A	Functional
“	“		Q167Q	Silent
“	“		R213 STOP	LOF
A-L	CC150	nd	R222F	Functional
“	CC286	-	R273C	LOF
“	“		Q317R	Functional
“	CC303	-	R306R	Silent
“	CC311	nd	N311N	Silent
“	“		E336E	Silent
“	CC325	-	N131N	Silent
“	CC326	+	None	Wild-type
	CC338	-	None	Wild-type

Mutations are designated with the wild-type amino acid, followed by the codon and the substituted amino acid. The Activity (i.e., functional consequence) of each mutation was determined by using the IARC (International Agency for Research on Cancer) TP 53 Mutation Database (http://www-p53.iarc.fr/) and scored as follows: Functional (change in aa sequence with equivalent function), Silent (no change in amino acid) or LOF (change in aa sequence with loss of function) (nd = not determined).

### The C-L group profile predicts a more favorable prognosis

Previous expression profiling studies of ovarian tumors have examined the overall survival (“Ovarian Cancer Prognostic Profile” or OCPP) [6] and the chemotherapeutic response (“Chemotherapeutic Response Profile” or CRP) [10] of ovarian cancer patients. To determine if these previously reported signatures could be used to make predictions regarding the C-L and A-L groups, we looked for overlap between OCPP and CRP with genes whose expression was significantly different between the C-L and A-L groups. While we found a few genes that overlapped with the CRP set including PDGFRα and RB1, the overlap between gene lists was not statistically significant (p>0.05). This lack of overlap may be due to the fact that all of the neo-adjuvant patients appeared to respond to chemotherapy clinically. For example, the tumors of all chemotherapy treated patients were similarly reduced in size and all treated patients displayed an equivalent reduction in levels of CA 125 prior to surgery (Table 5). Also relevant is the fact that there was no significant difference in the age of patients in the C-L and A-L group (Table 1). In addition, our microarray analysis was conducted after chemotherapy treatment, and thus subsequent to any differential selection imposed by the neo-adjuvant treatment. In this regard, it is relevant to note that none of the untreated cancer patient samples (CA) clustered within the A-L group, indicating that the cell composition of the neo-adjuvant cancer patient samples (CC) displaying the A-L group expression pattern was most likely selectively amplified in response to the chemotherapy.

**Table 5:** CA125 values of chemotherapy treated patients at various time points prior to surgery.

CLUSTER	PATIENT(age)	CA 125 Levels					
		(months prior to surgery)					
		(5 months)	(4 months)	(3 months)	(2 months)	(1 month)	Day of Surgery
C-L							
	CC187(53)	ND	2347	2204	280	30	24
	CC199(69)	ND	ND	6829	675	ND	66
	CC255(57)	696	465	ND	117	ND	41
	CC259(58)	ND	4626	ND	47	ND	23
	CC269(68)	ND	681	ND	39	ND	20
	CC272(83)	173	ND	228	ND	116	25
	CC312(64)	ND	2683	1831	231	35	18
A-L	CC150(65)	10,284	942	117	33	25	22
	CC286(52)	ND	ND	286	ND	13	30
	CC303(44)	ND	ND	352	ND	37	25
	CC311(51)	3576	ND	2123	1541	187	45
	CC325(75)	1300	974	131	ND	59	38
	CC326(72)	2450	ND	ND	651	ND	125
	CC338(62)	ND	516	264	46	8	14

The time point most distant from day of surgery reflects the CA 125 value prior to the administration of the first of three to four chemotherapy treatments. The results demonstrate that all patients responded positively to the chemotherapeutic treatment (ND = not determined).

In contrast, when the list of genes altered between our C-L and A-L sample groups was compared to the OCPP, we observed a significant overlap (p = 0.005 by hypergeometric distribution; Table 6). Interestingly, 21/23 (91%) of the overlapping genes displayed changes in expression predictive of a favorable outcome for the C-L group of samples.

**Table 6:** Genes displaying significantly altered expression between A-L and C-L samples that overlap with previously published Ovarian Cancer Prognostic Profile (OCPP) [6] or the Chemotherapy Response Profile (CRP) [10].

*Probe ID*	*Symbol*	*Fold Change*	*Overlap*	*Favorable*
39660_at	DEFB1	15.8	OCPP	Yes
1585_at	ERBB3	6.9	OCPP	Yes
38143_at	KLK7	5.2	OCPP	Yes
36499_at	CELSR2	3.8	OCPP	Yes
38160_at	LY75	3.3	OCPP	Yes
37956_at	ALDH3B2	3.3	OCPP	Yes
40030_at	PRKY	2.5	OCPP	Yes
311_s_at	—	2.2	OCPP	No
36577_at	PLEKHC1	−2.1	OCPP	Yes
33440_at	TCF8	−2.1	OCPP	Yes
34320_at	PTRF	−2.5	OCPP	Yes
39066_at	MFAP4	−2.5	OCPP	Yes
36119_at	CAV1	−2.6	OCPP	Yes
38653_at	PMP22	−2.6	OCPP	Yes
34303_at	FLJ90798	−2.7	OCPP	Yes
34802_at	COL6A2	−2.8	OCPP	Yes
1771_s_at	PDGFRB	−3.2	OCPP	Yes
39864_at	CIRBP	−3.2	OCPP	Yes
36993_at	PDGFRB	−3.2	OCPP	Yes
1319_at	DDR2	−3.5	OCPP	Yes
37221_at	PRKAR2B	−3.9	OCPP	No
743_at	NAP1L3	−4.7	OCPP	Yes
35168_f_at	COL16A1	−6.0	OCPP	Yes
39419_at	SPAG9	−2.4	CRP	N/A
33922_at	PRDM2	−2.2	CRP	N/A
35735_at	GBP1	2.1	CRP	N/A
1105_s_at	TRBV21-1	2.2	CRP	N/A
2044_s_at	RB1	2.5	CRP	N/A
2067_f_at	BAX	2.6	CRP	N/A
915_at	IFIT1	3.0	CRP	N/A
41827_f_at	LOC91316	3.4	CRP	N/A
33274_f_at	IGLC-1	4.1	CRP	N/A
33273_f_at	IGLC-1	4.4	CRP	N/A
406_at	ITGB4	6.0	CRP	N/A
2027_at	S100A2	9.0	CRP	N/A

Fold change is positive if higher in carcinoma-like samples. Favorable indicates whether the directional fold change predicts favorable or unfavorable outcome for the C-L samples.

### C-L group patients survive longer than A-L group patients

**Figure 7:**
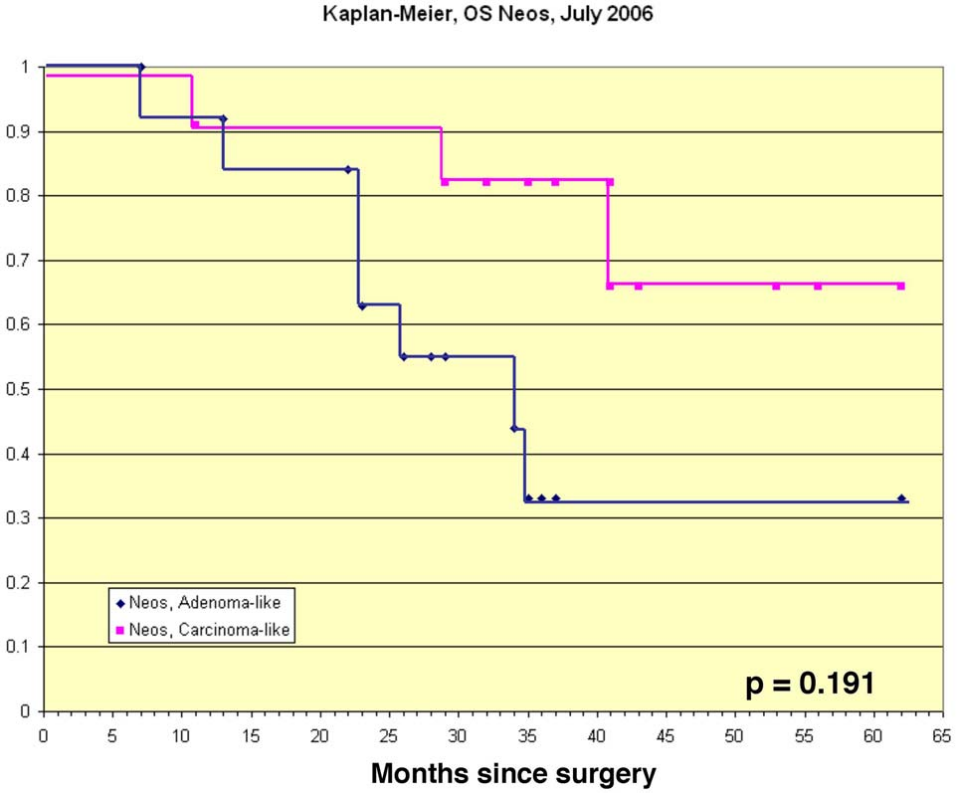
Cumulative survival curves of ovarian carcinoma patients treated with neo-adjuvant chemotherapy. Seventy percent of neo-adjuvant cancer patients whose tissue gene expression profiles clustered with those of cancer patients not pre-treated with chemotherapy have survived five years after surgery. In contrast, only thirty percent of patients whose expression arrays clustered with benign adenoma patients prior to surgery survived five years after surgery. Although the trends are clear, because of the limited sample size of the cohort of patients for which follow-up data were available (A-L group n = 13; C-L group n = 11), the differences are marginally insignificant (p<0.191, logrank test). The survivorship data used in the computation of the Kaplan-Meier plot can be found in the supplementary material (Figure S2).

To test the prediction that C-L group patients will have a more favorable outcome than A-L group patients, we examined the relative survivorships of the 24 CC patients in these two groups over the 5 years that have elapsed since the initiation of our study. We employed the Kaplan-Meier method [30] to model the time to death of CC patients clustering in the A-L and C-L groups. Consistent with the prediction based upon the overlap with the OCPP profile, the neo-adjuvant patients whose expression array profile clustered in the C-L group have had a more favorable outcome than those whose profile clustered with the A-L group (Figure 7).

Although the trends are clear, because of the limited sample size of the cohort of patients for which follow-up data were available (A-L group n = 13; C-L group n = 11), the differences are marginally insignificant (p<0.191, logrank test). Nevertheless, seventy percent of neo-adjuvant cancer patients whose tissue gene expression profiles clustered with the C-L group have survived five years after surgery. In contrast, only thirty percent of patients whose expression arrays clustered with the A-L group survived five years after surgery.

## Discussion

Our results indicate that the neo-adjuvant patient samples (CC) that cluster with the untreated cancer samples (i.e., the C-L group), are preferentially associated with LOF mutations in the *p53* gene while few such mutations are associated with the CC samples clustering with the non-malignant adenomas (the A-L group). The fact that only one A-L group tumor is associated with a *p53* mutation, that the A-L group tumors display reduced BAX expression and increased Bcl-2 levels suggest that they are capable of undergoing cell cycle arrest (G1/S checkpoint) while the majority of those clustering with the malignant carcinomas (C-L group) may not. The C-L group patients display increased expression of genes typically associated with increased proliferation and preferential association with LOF mutations in the *p53* gene.

The fact that nearly all of the A-L group tumors are wild-type for *p53* implies that they are able to arrest in response to DNA-damage. Cell cycle arrest provides an opportunity for DNA repair, but cells that have accumulated irreparable damage are channeled into the apoptotic pathway [31]. While the DNA repair process is focused on the damage induced by the chemotherapeutic treatment, it does not address the inherited defects that rendered cells malignant in the first place. We hypothesize that at least some of the DNA damaged cells that undergo cell cycle arrest and are successfully repaired, (i.e., not channeled into the apoptotic pathway) may retain the malignant genotype and resume growth after treatment. We hypothesize that it is these cells that may contribute to tumor recurrence.

In contrast, we propose that our C-L group tumors that are associated with *p53* LOF alleles proceed through the cell cycle in the presence of significant DNA damage leading to eventual death either by way of a *p53* independent apoptotic pathway or by mitotic catastrophe [32]. Consistent with this model, it has been previously shown that loss of *p53* function in human ovarian cancer cells results in an increase in cisplatin cytotoxicity with a correlated loss of G_1_/S checkpoint control [33]. Also consistent is the recent observation that head and neck squamous carcinoma cells mutant for *p53* display increased susceptibility to cisplatin-induced apoptosis [34].

One of the evolved objectives of the DNA damage response in biological systems is the repair of the damage and the resumption of cellular growth and replication. In contrast, the clinical objective of DNA damaging chemotherapy is cell death. Thus, any potential that cancer cells may harbor to repair chemotherapy-induced DNA damage can be viewed as counter productive from the clinical perspective. While it has been reported that p53 may enhance chemotherapeutic treatment in some cellular contexts [35], [36], our results suggest that *p53*-mediated cell cycle arrest/DNA damage repair is serving as a barrier to the desired clinical outcome of DNA damaging chemotherapy in ovarian cancer. For this reason and others [37]–[39], the potential benefit of inhibiting *p53* in conjunction with DNA damaging agents in the treatment of ovarian and perhaps other malignancies may be a protocol worthy of further consideration. Studies are currently underway in our laboratory to test the relative effectiveness of chemotherapeutic agents in p53-inhibited cells vs. controls.

## Materials and Methods

### Tumor samples and RNA isolation

A set of 43 ovarian tumors was obtained from the Ovarian Cancer Institute (Atlanta): 10 benign serous cystadenomas, 9 adenocarcinomas from patients not pre-treated with chemotherapy prior to surgery (7 serous papillary, Stages II, III and IV; 2 endometriod, Stages I and III and 24 adenocarcinomas from patients having neo-adjuvant therapy (22 serous papillary, Stages III and IV; 1 endometriod, Stage III; 1 undifferentiated, Stage III). The average age of the patients participating in the study on the day the samples were collected was 61 (range 41–84). This study was approved by the Institutional Review Boards of Georgia Institute of Technology and Northside Hospital, (Atlanta) from which the samples originally were obtained. Tissue was collected at the time of surgery and preserved in RNAlater (Ambion, Austin, TX) within one minute of collection. Linear polyacrylamide (5 ul) was added to ∼50 mg of tumor sample and homogenization was carried out on ice in 1.5 ml Trizol (Invitrogen, Carlsbad, CA) with a polytron homogenizer for 30 seconds. RNA was isolated from crude homogenate according to the manufacturer's protocols (Trizol) and further enriched using an RNEasy column (Qiagen, Valencia, CA).

### Microarray hybridization

Biotinylated target cRNA was generated and cleaned by phenol/chloroform extraction/ethanol precipitation according to established protocols (Afftmetrix, Santa Clara, CA). *In vitro* transcription of the cDNA using the High Yield RNA Transcript Labeling Kit (Enzo, Farmingdale, NY) yielded 50–100 ug of biotin labeled cRNA target. The cRNA was fragmented to a length of 20–200 bp and hybridized to the Affymetrix HG-U95Av2 array for 16 hrs at 45 C. Hybridized arrays were washed, stained and scanned according to established protocols (Affymetrix).

### Microarray Data Analysis

Thirteen patient array profiles from our previously published pilot study [40] were combined with 30 previously unpublished arrays for this analysis. Gene expression data from 12,625 probe sets on the HG-U95Av2 GeneChips were normalized, using GCRMA normalization with GeneTraffic Software (Iobion, La Jolla, CA). After data normalization, genes with uniformly low expression were removed from consideration, leaving 9,106 probe sets for analysis using Significance Analysis of Microarrays (SAM 2.23) software [11]. Relevant parameters for the SAM analysis were: Imputation engine, 10-Nearest Neighbor; Number of Permutations, 500; RNG Seed, 1234567; Delta, 1.105; Fold-Change, 2.0; False Discovery Rate<1%. Normalized expression data from the 1,527 significant probe sets were analyzed by a two-dimensional hierarchical clustering using Cluster 3.0 [41] and Java Treeview 3.0 [42]. Both genes and arrays were normalized, median-centered, and hierarchically clustered using unweighted averages and ordered using average Euclidian distance. Analysis of gene sets for enrichment in GO functional annotations, were performed using GOstat [12].

For K-nearest neighbor (KNN) prediction, the GCRMA normalized microarray data were analyzed with GenePattern 2.0 software [43] and both the KNN cross-validation and KNN class prediction modules were used (KNN = 3). All 9,106 probe sets that had detectable expression were available for inclusion in each model. For these analyses 30 genes (or features) were used for each of the KNN prediction models. In the Leave-one-out cross-validation KNNxVal models, 43 models were generated using 42 samples to predict the class of the sample that was left out. For the four-fold cross validation, the set of 43 samples was randomly divided into a training set of 2/3 of the samples (29 training samples) and a test set of the remaining 1/3 of the samples (14 samples). This random selection was done four times, resulting in four training sets, four test sets, and four KNN models. Thus, four test sets of 14 samples resulted in a total of 56 predictions.

### Pathway and Profile Analyses

Lists of probe sets that were predictive by KNN analysis and lists of significantly different genes from SAM analysis were submitted to pathway analysis using Ingenuity Pathway Analysis (IPA) software (http://www.ingenuity.com). This software generates networks of genes using interactions present in the Ingenuity Pathways Knowledge Base that are derived from manually curated and extracted interactions from the published scientific literature. It also identifies overrepresented biological functions and canonical pathways based on the GO annotations of each gene and manually curated canonical pathways. The significance value associated with Functions and Pathways is a measure for how likely it is that a gene list participates in a function. The significance is expressed as a p-value, that is calculated using the right-tailed Fisher's Exact Test by comparing the number of user-specified genes of interest that participate in a given function or pathway, relative to the total number of occurrences of these genes in all functional/pathway annotations stored in the Ingenuity Pathways Knowledge Base.

Downstream targets of p53 were identified by merging interaction networks identified by IPA and generating all direct transcriptional links from p53 to genes in the network. Relationships were examined manually in the Ingenuity Pathways Knowledge Base to determine if target genes were activated or repressed by p53. All relationships were based on published literature references.

For the gene overlap profile analysis, the intersection of the 115 probes in the OCPP with 1,527 probes significantly different between A-L and C-L samples was determined to include 23 probes. The probability that the intersection of these two groups would include 23 probes given a total of 12,626 probes on the HG-U95Av2 array was computed by the hypergeometric distribution using the R statistical programming language (http://www.R-project.org) and the p-value (p = 0.005) was computed.

### Quantitative RT-PCR

Total RNA (2 ug) from ovarian tissue was converted to cDNA using Superscript III (Invitrogen) primed with random hexamers under conditions described by the supplier. cDNA from this reaction was used directly in the qRT-PCR analysis. Gene specific primers for three genes (BAX: Forward 5′ GCTGTTGGGCTGGATCCAAG 3′, Reverse 5′ TCAGCCCATCTTCTTCCAGA 3′;CCND1: Forward 5′ ACGAAGGTCTGCGCGTGTT 3′, Reverse 5′ CCGCTGGCCATGAACTACCT 3′, BCL2: Forward 5′ CTGGTGGGAGCTTGCATCAC 3′, Reverse 5′ ACAGCCTGCAGCTTTGTTTC 3′) were synthesized (Integrated DNA Technologies, Coralville, IA). The mRNA levels of the three genes were measured in 2 malignant and 2 benign tumor samples on the DNA Engine Opticon 2 System (MJ Research). PCR was performed using the Cybergreen PCR MasterMix (Applied Biosystems) according to the manufacturer's protocols. Using GAPDH as a control, the expression levels of BAX, CCND1 and BCL2 were calculated according to the 2^−ΔΔCt^ method [44]. The Ct values of triplicate RT-PCR reactions were averaged for each gene in each cDNA sample. For each tissue sample assayed, the level of gene expression for the gene of interest (BAX, CCND1 and BCL2) was calculated against that of the housekeeping gene (GAPDH). Each Δ Ct value was normalized to the lowest expressing sample to obtain the ΔΔ Ct value. The standard deviation was calculated for samples within each tissue class.

### Histopathologic and immunohistochemical evaluation

Histologic slides were studied to confirm histopathologic diagnosis of cancer, and representative formalin fixed, paraffin embedded tissue blocks were selected for p5*3* immunohistochemical analysis. Three tissue cores measuring 1.0 mm from each block were obtained for tissue microarray (TMA) (Beecher Instruments, Sun Prairie, WI). The TMA slides were deparaffinized through xylene changes and rehydrated in increasing gradations of alcohol. Antigen retrieval was performed using Biocare Medical Decloaking Chamber with DAKO Target Retrieval Solution, pH 6.0. The slides were washed in TBS buffer and loaded onto the DAKO universal autostainer. Slides were blocked in 3% hydrogen peroxide followed by incubation of p53 (clone DO-7, DAKO Corp., Carpinteria, CA) primary antibody at 1:80 dilution for 30 minutes. The DO-7 detects both wild-type and mutant p53 protein. Visualization was achieved utilizing DAKO Envision+ with DAB. The slides were counterstained with hematoxylin and the immunohistochemical staining pattern was assessed using a Nikon microscope. The staining was assayed using a semi-quantitative scoring from 0 to 3+ (0: no staining, 1+: <10% nuclei staining, 2+: 11–50% nuclei staining, 3+: >50% nuclei staining). For the purpose of recording the results, intensity of staining was not taken into consideration, but generally the intensity correlated with the percentage of nuclei staining positive (strongest in 3+ and the weakest in 1+). Samples displaying a ≥ 2+ signal in>50% of cells examined were scored as positive.

### p53 sequence analysis

RNA samples extracted from adenocarcinomas removed from 14 neo-adjuvant patients, adenocarcinomas from 5 patients not pre-treated with chemotherapy prior to surgery, and benign adenomas from 6 patients were used to synthesize cDNA (SuperScript III, Invitrogen). PCR (polymerase chain reaction) amplification was performed to amplify the exons 5-10 of the *p53* gene where the majority of the functionally important mutations in human cancer have been mapped [45]. The primer used for the forward direction was 5′-GCA CGT ACT CCC CTG CCC TCA A-3′ [35]. The primer used for the reverse direction encompassed the 3′-end of exon 10 minus the stop codon, 5′-GTC TGA GTC AGG CCC TTC TGT C-3′ [27]. A 768 bp PCR product was generated using the following conditions: 1) 95 C, 5 min; 2) 95 C, 40 sec; 3) 59 C, 40 sec; 4) 72 C, 1 min; 5) repeat steps 2–4 30×; 6) 72 C, 5 min; 7) 4 C hold. Amplified PCR products were cloned into TA vectors using the TOPO TA cloning kit (Invitrogen), and the resulting isolated plasmids analyzed using 1% agarose gels to check for PCR insertions. Sequencing of each PCR reaction was performed in both directions using the M13 Forward and M13 Reverse primers provided with the TOPO TA cloning kit. Each PCR experiment was repeated independently at least twice. DNA sequencing was carried out at the University of Georgia DNA sequencing facility (Athens, GA). Sequences were aligned and analyzed for mutations using MacVector. The functional significance of identified *p53* mutations was scored by using the IARC (International Agency for Research on Cancer) TP 53 Mutation Database (http://www-p53.iarc.fr/).

## Supporting Information

Figure S1 Unsupervised hierarchical clustering of the entire gene expression pattern of all 9,106 expressed probe sets detected on the HG-U95Av2 GeneChip in 43 ovarian tumor samples. Samples beginning with AD are adenomas, with CA are carcinomas, and CC are patients pre-treated with chemotherapy. Samples divided into two major clusters termed adenoma-like and carcinoma-like.
(10.15 MB TIF)

Figure S2 Survivorship data of A-L and C-L group cancer chemo patients (CC) used in the Kaplan-Meier analysis (See Figure 7).
(8.02 MB TIF)
