# Supplementary figures and images for: Evidence that p53-Mediated Cell-Cycle-Arrest Inhibits Chemotherapeutic Treatment of Ovarian Carcinomas
Source: PLoS One. 2007 May 16;2(5):e441. doi: 10.1371/journal.pone.0000441 (PMC1859837; doi:10.1371/journal.pone.0000441)

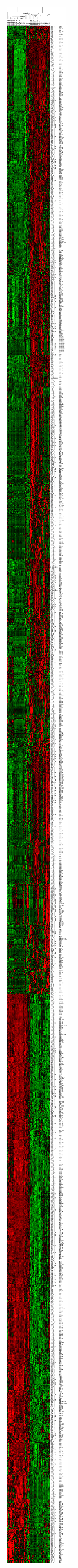

Supplement: Figure S1 — Unsupervised hierarchical clustering of the entire gene expression pattern of all 9,106 expressed probe sets detected on the HG-U95Av2 GeneChip in 43 ovarian tumor samples. Samples beginning with AD are adenomas, with CA are carcinomas, and CC are patients pre-treated with chemotherapy. Samples divided into two major clusters termed adenoma-like and carcinoma-like. (10.15 MB TIF) [file pone.0000441.s001.tif]

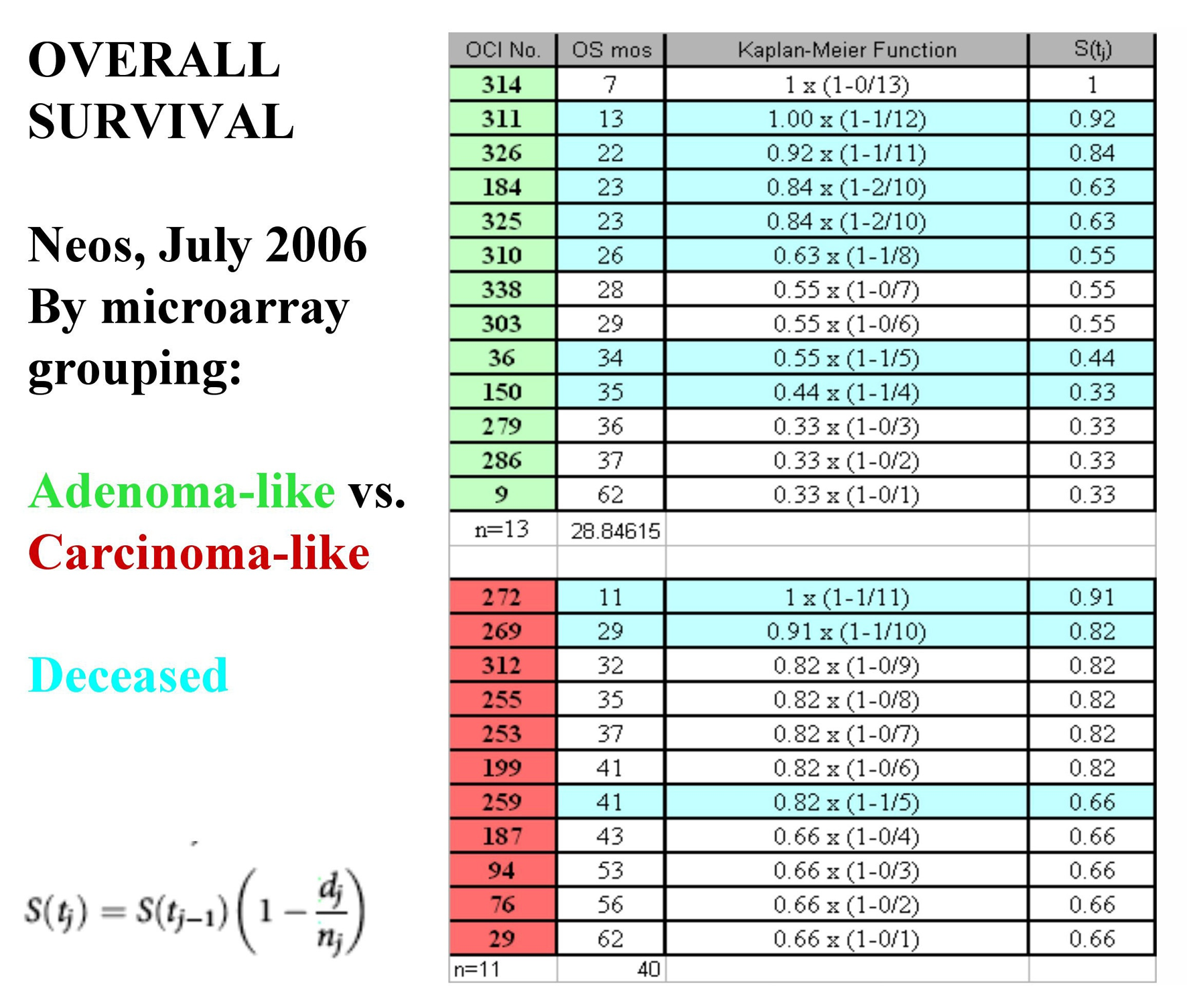

Supplement: Figure S2 — Survivorship data of A-L and C-L group cancer chemo patients (CC) used in the Kaplan-Meier analysis (See Figure 7). (8.02 MB TIF) [file pone.0000441.s002.tif]
